# Introducing the subcutaneous depot medroxyprogesterone acetate injectable contraceptive via social marketing: lessons learned from Nigeria's private sector^☆^

**DOI:** 10.1016/j.contraception.2018.07.005

**Published:** 2018-11

**Authors:** Jenny Liu, Eric Schatzkin, Elizabeth Omoluabi, Morenike Fajemisin, Chidinma Onuoha, Temitope Erinfolami, Kazeem Ayodeji, Saliu Ogunmola, Jennifer Shen, Nadia Diamond-Smith, Maia Sieverding

**Affiliations:** a Institute for Health and Aging, Department of Social and Behavioral Sciences, School of Nursing, University of California, San Francisco, 3333 California Street Suite 340, San Francisco, CA 94118, USA; b Akena Associates, House 20 F Road, CITEC Mbora Mount Pleasant, Off Jabi Airport Road, Abuja, Nigeria; c DKT Nigeria, 42 Montgomery Road, Lagos, Nigeria; d Institute for Health Policy Studies, School of Medicine, University of California, San Francisco, 3333 California Street, Suite 266D, San Francisco, CA 94118, USA; e Global Health Sciences, Department of Epidemiology and Biostatistics, School of Medicine, University of California, San Francisco, 550 16th Street Mission Hall, San Francisco, CA 94158, USA; f Department of Health Promotion and Community Health, Faculty of Health Sciences, American University of Beirut, PO, Box 11-0236, Riad El-Solh, Beirut, 1107 2020, Lebanon

**Keywords:** Injectable contraceptive, Subcutaneous depot medroxyprogesterone acetate, Nigeria, Social marketing, Private sector, Community-based distribution

## Abstract

**Objectives:**

The subcutaneous depot medroxyprogesterone acetate (DMPA-SC) injectable contraceptive was introduced in South West Nigeria in 2015 through private sector channels. The introduction included community-based distribution and was supported by a social marketing approach. From program monitoring and evaluation, aimed at understanding performance, market reach and other process measures, we identify lessons learned to inform future scale-up efforts.

**Methods:**

We synthesized the findings from a core set of key performance indicators collected through different methods: (1) implementer performance indicators, (2) phone survey of DMPA-SC users (*n*=541) with a follow-up after 3 months (*n*=342) and (3) in-depth interviews with 57 providers and 42 users of DMPA-SC.

**Results:**

Distribution of DMPA-SC to private providers was concentrated in states with large urban populations. A shift toward focusing on high-volume family planning facilities coincided with a rapid increase in distribution in late 2016. Users reached in the phone survey were generally older and married with children; few were under age 25. Users and providers reported favorable opinions of DMPA-SC. Many users reported choosing DMPA-SC due to recommendations from providers and friends, and the hope of experiencing reduced side effects compared to other methods. While users reported positive experiences interacting with community-based distributors, the delivery model encountered a number of challenges — high turnover, low motivation, lack of an appropriate compensation package and logistical costs — and was ultimately disbanded.

**Conclusions:**

In the DMPA-SC introductory program in Nigeria, distribution was amplified when focused on high-volume contraceptive providers. Although community-based distribution can be one effective service delivery model for reaching underserved populations, more consideration for balancing cost recovery and public health goals through private sector approaches are needed in the context of South West Nigeria. Additional communications and outreach efforts are needed to reach younger, unmarried users with contraceptive services.

## 1 Introduction

Reducing unmet need for modern contraception remains a priority in global public health [1]. In Nigeria, Africa's most populous country, unmet need remains high as many women continue to face barriers to accessing contraception, including lack of trained health providers, provider biases against contraceptive use among certain subpopulations of women, lack of confidentiality, and cultural and religious opposition [2], [3]. The modern contraceptive prevalence rate (mCPR) among all married women increased from less than 10% in 2013 to 16.0% in 2016 [4], [5]. The Government of Nigeria, in collaboration with both public and private sector partners, has pledged to achieve an mCPR of 27% by 2020 [6], [7]. To accelerate progress toward this goal, subcutaneous depot medroxyprogesterone acetate (DMPA-SC, brand name Sayana Press) was introduced to the contraceptive method mix in Nigeria — first through private sector providers via social marketing in 2015 and later through public sector providers in late 2016. Nigeria's total market approach to the introduction of DMPA-SC departed from the more controlled research or pilot launch conditions implemented in other countries [8], [9], [10]. To monitor and evaluate the private sector launch, a suite of data collection activities was designed to generate timely information for program course correction and to document insights capable of informing scale-up efforts. This paper presents synthesized findings and lessons learned from the monitoring and evaluation (M&E), many of which highlight important considerations for future efforts to introduce and expand the use of DMPA-SC in the private sector in Nigeria and beyond.

### 1.1 The introduction of DMPA-SC in Nigeria

DMPA-SC was introduced to Nigeria in January 2015 by DKT Nigeria, a nonprofit organization specializing in contraceptive social marketing, a strategy that leverages existing market infrastructure, incentives and methodologies to expand access to contraceptive services and products. For a detailed description of the organization, its operations and the DMPA-SC program, please see the Supplementary materials. DKT Nigeria's contraceptive social marketing aimed to reduce unmet need for contraception and increase access to reproductive health services by: (1) ensuring wide availability of high-quality, affordable reproductive health products through private sector supply chains and (2) creating demand through integrated, evidence-based behavior change campaigns. For the DMPA-SC introduction program, supply-side components included (1) medical detailing to service providers including a transition to targeting high-volume contraceptive providers, (2) utilizing existing wholesale distribution channels and linkages with professional health worker associations, (3) offering provider trainings and (4) establishing a new community-based distribution (CBD) delivery model. Demand-side components included (1) using traditional mass media approaches through television, radio, and posters and banners hung at providers' facilities; (2) leveraging digital and online media; (3) creating an independent brand (i.e., honey and banana) focusing on reproductive health and (4) offering an SMS message reminder service to users to facility timely reinjections. These components are described below.

#### 1.1.1 Supply-side program features

Though there were a small number of small-scale DMPA-SC pilot projects in Nigeria, DKT Nigeria remained the only large procurer and distributor of DMPA-SC until public sector provision began in late 2016. DKT Nigeria's DMPA-SC program initially focused efforts in seven South West states (Lagos, Oyo, Ogun, Osun, Ondo, Ekiti and Kwara), distributing the product through existing private sector providers [e.g., health facilities and drug shops] and established distribution channels (i.e., wholesalers and medical professional associations), through which a large proportion of contraceptive users in Nigeria obtain their methods [4]. Each DMPA-SC unit (i.e., single contraceptive injection dose) was sold to providers for 250 Naira (~US$1.25) with a recommended retail price of 500 Naira (~US$2.50); providers could also charge an additional fee for administration. Medical sales representatives, DKT Nigeria's sales force tasked with medical detailing to health providers and responsible for last-mile distribution of contraceptives, were trained and deployed to directly engage with health providers on DKT's product portfolio, including DMPA-SC. A sales database was instituted in October 2015 to facilitate real-time tracking of all product sales to service providers and provide data for operational improvements.

In August 2016, the DKT Nigeria reoriented the DMPA-SC sales operations around a “hotspot” approach, which identified and focused distribution to high-volume reproductive health service establishments. Rewards of equipment or supplies were offered for different quantities of bulk purchases (e.g., 100 units, 200 units, or 500 units) of DMPA-SC and other reproductive health products offered by DKT Nigeria. Rewards were linked to the value of contraceptives purchased and not to client utilization, and each reward tier included quantities for different products (e.g., IUDs, pills, implants) to ensure that these facilities could offer a variety of contraceptive products. DKT Nigeria routinely provided facility-based DMPA-SC trainings for new hotspot facilities.

DKT Nigeria also offered service providers training on counseling for and administration of DMPA-SC. Training content used PATH's training curriculum for DMPA-SC but was adapted for Nigeria to include a general overview of contraceptive methods and counseling and specific information on DMPA-SC (e.g., unique features, comparison with other instramuscular injectables, benefits, side effects and their management) including an injection practicum [11], [12]. Although trainings of private sector providers were held consistently throughout the project period, DKT Nigeria adjusted the training recruitment approach to better target only providers who were already interested or involved in reproductive health service provision. This was a result of early experiences with trainees who were discovered to be disinterested in contraception when training was offered to any provider invited by sales representatives. Coinciding with the launch of the hotspot program, DKT shifted from a centrally located training venue with participants brought from many facilities to facility-based trainings held only for relevant facility employees. This shift helped improve the targeting and efficiency of trainings and ensured that more than one health worker at each facility could provide contraceptive services.

An additional program feature was the development of a private sector CBD channel, directly managed by DKT Nigeria, to proactively offer more personalized, accessible contraceptive service in community settings (e.g., at clients' homes, in marketplaces). One aspirational goal of this CBD approach was to reach underserved women with unmet need for contraception (e.g., from rural areas, younger women). Beginning in August 2015, a cadre of trained and licensed Community Health Extension Workers (CHEWs) was recruited and branded as “DKT Bees.” Bees went into communities and offered contraceptive services, including counseling; pregnancy testing; and provision of condoms, oral pills or DMPA-SC.^1^ Bees were trained to refer clients to a facility for longer acting contraceptives such as implants or IUDs, which were beyond the Bee scope of practice. The recommended retail price for DMPA-SC from Bees was also 500 Naira (~US$2.50), inclusive of administration. Bee performance was measured based on the quantity of units administered to women in the communities where they were deployed. As financial incentives, Bees were permitted to keep the profit markup on all products sold, along with receiving regular allowances for transportation and communications.

While CHEWs were able to administer injections by virtue of their licensing [13], conducting proactive CBD was a new model of service delivery for this cadre. Even though CHEWs were as originally designed and trained for CBD work, facility-based service provision had evolved to become their service delivery norm over time [14]. As such, many Bees recruited early in the program were unfamiliar with and unprepared for the rigors of community engagement and proactive outreach. To address high turnover and initial low performance of many recruited Bees, DKT Nigeria adjusted the operational model to include more rigorous screening for highly-motivated candidate — those better prepared for the travel and interpersonal communications of community outreach — along with additional training for Bees on business management and customer engagement skills. DKT Nigeria also launched a form of supportive supervision by DKT-employed nurses beginning late 2015. DKT Nigeria again restructured the Bee program in Quarter 2 2016 in response to some misuse of transportation and communications allowances: Bees would now receive earnings of 50% of all products sold without further stipends. Despite these adjustments, DKT Nigeria ended the Bee program in late 2016 due to continued low performance.

#### 1.1.2 Demand-side program features

Due to regulatory restrictions on the marketing of prescription medicines, direct-to-consumer marketing for DMPA-SC was not allowed. Thus, communications could not specifically refer to DMPA-SC as a product or its brand name, an additional challenge for creating awareness for the product's availability in Nigeria. DKT Nigeria therefore launched demand generation for general reproductive health services in September 2016. Activities included programs that educated audiences about contraceptive options, addressed myths and misconceptions, and discussed common side effects and their management. Programs were aired on traditional media (e.g., TV, radio) and digital and online media, including a dedicated website (honeyandbanana.com) and social media platforms such as Facebook, Instagram and Twitter. Many of these activities were launched in rapid succession, and the full media outreach efforts were in place by early 2017.

To support client compliance with reinjections, DKT Nigeria also established a free, SMS-based reminder alert service beginning late 2015. To enroll users, DKT Nigeria sales representatives asked providers to inform DMPA-SC users of the service. A subscription code was also affixed to each DMPA-SC unit package. Once registered, users received automated text message alerts timed for the 3-month reinjection window.

### 1.2 Monitoring and evaluation

Because this program was the first large-scale introduction of DMPA-SC in Nigeria, M&E were implemented alongside program operations to understand performance, market reach and other process measures that could be used to continuously improve the program. A mixed-methods approach to data collection was designed to monitor and assess a set of Key Performance Indicators (KPIs) for the program. A primary focus of data collection was to generate information about the CBD model, both because the model delivered contraceptive services outside traditional health system channels and because the approach leveraged the recently expanded scope of work for CHEWs to provide injectables [5].

## 2 Methods

### 2.1 KPIs

The M&E were designed around a core set of KPIs, defined at the outset of the program to track progress toward DKT Nigeria and donor objectives. The KPIs focused on four key areas of program delivery: (1) product distribution, (2) provider training and CBD model, (3) users' profiles and (4) provider and user experience with DMPA-SC. KPIs were a combination of indicators used in other DMPA-SC pilot programs, for comparability, and program-specific indicators to monitor unique features of the Nigeria introduction (see Supplementary materials for a full list) [15]. Three sources of data were used to calculate KPIs:

1. Program documents, reports and internal sales data extractions, supplemented with direct communications with program staff for further clarification and context;
2. A phone survey administered to DMPA-SC users to collect information on users' sociodemographic profiles and their experiences with obtaining DMPA-SC;
3. In-depth interviews (IDIs) with both DMPA-SC users and providers to gain a richer understanding of their receptivity toward the product, challenges encountered and concerns about continued use or provision (i.e., aspects that could not be adequately captured in the short, quantitative phone survey).

Each of these methods is described below. Data collection efforts were limited to the seven South West states where product distribution was first introduced.

KPIs were descriptively analyzed for variation across time, states and delivery channels. A subset of KPIs (see Supplementary materials, Table S2 Objective 1) was measured against target outputs set at the beginning of the program. In alignment with the secondary objectives of the DMPA-SC introduction, we also examined program reach for four user segments of interest:

1. New users of modern contraception, defined as women who had never used a modern method before.
2. Current users switching from noninjectable modern methods.
3. Current users switching from other injectables (e.g., Depo Provera, Noristerat).
4. Young (age <25) or unmarried users.

The first three categories, consisting of new users, current users switching from noninjectable modern methods and current users switching from other injectables, are mutually exclusive; the last category for young or unmarried women is separately identified based only on these demographic criteria and could include both new and current users of contraceptives.

### 2.2 Program documents, reports and internal sale data extractions

Raw data inputs on DMPA-SC distribution, provider training and the CBD program were submitted by DKT Nigeria on a regular basis and then collated and aggregated by the M&E team. Much of the program data were extracted from the organization's automated sales data management system implemented in October 2015. Distribution data reflected sales to providers and wholesale channels; information about end-users of contraceptive products was not available from program records, including product uptake and administration. Monthly sales orders were disaggregated by facility, enabling us to calculate the percentage of facilities placing reorders of DMPA-SC by month — a proxy for DMPA-SC administration to clients. The number of providers trained was disaggregated by state and type of provider (e.g., doctor, nurse, pharmacy, Bee). An Excel-based tool was used to organize these data by month and filled out on a quarterly basis. Ad hoc personal communications with different program staff members were held to clarify discrepancies and ambiguities, as well as provide additional context for interpreting data inputs.

### 2.3 External data collection

**Fig. 1:**
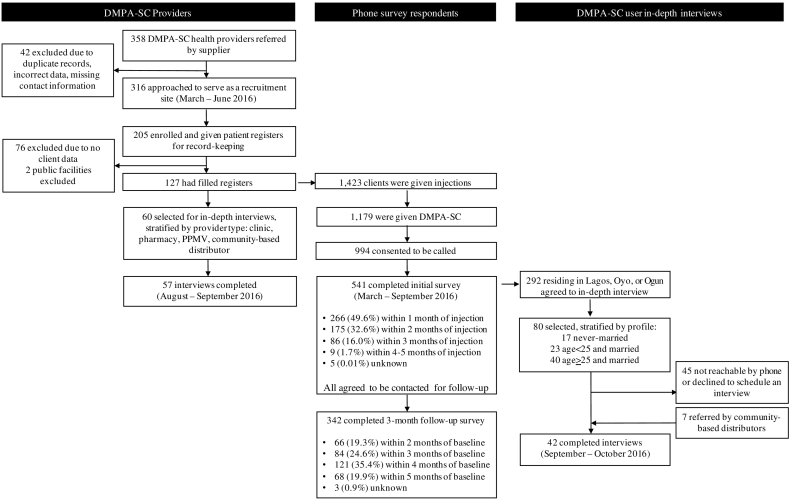
Recruitment flowchart. Of 358 DMPA-SC health providers referred to the research team by DKT Nigeria, 205 providers were enrolled into the study as user recruitment sites and asked to keep patient registers for women obtaining injectable contraceptives. Of these, 127 provided register data, and 60 providers were later selected for in-depth interviews, stratified by provider type, of which 57 were completed. Of the 1423 users of injectable contraceptives recorded in patient registers, 1179 had purchased DMPA-SC, and 994 consented to be called for a phone survey. Of 541 women completing the initial survey, 342 (63.2%) completed a follow-up survey timed for after they were due for reinjection. In addition, 80 women agreeing to an in-person interview in the phone survey were selected for individual in-depth interviews. After an additional 7 women were referred to the research team for in-depth interviews, 42 interviews were completed.

To better understand user profiles and user and provider experiences with DMPA-SC, we undertook additional primary data collection. In the absence of a comprehensive registry of private sector facilities in the target states, we used DKT Nigeria's sales database to recruit providers offering DMPA-SC and, through them, product users. Fig. 1 details the recruitment flow for the M&E activities, and the sampling methodology and data collection activities are described in detail in the Supplementary materials.

In summary, DKT Nigeria provided a list of 358 providers who purchased DMPA-SC between October 2015 (the start of organizational electronic data capture) and February 2016 when recruitment for research began. This list included health facilities (i.e., hospitals, clinics, and maternity homes), drug retail outlets and DKT Bees. Notably, this set of providers was identified prior to the beginning of the hotspot program, and thus, de novo data reflect experiences with DMPA-SC from providers and users early in the DMPA-SC program. From March to June 2016, the study team visited each of the 316 providers with enough information to be contacted or located. After identifying the person in charge, enumerators explained the purpose of the study and its procedures, and obtained verbal informed consent to participate as a recruitment site. For the 205 providers consenting to participate, one provider at each facility who was in charge of delivering contraceptive services was asked to keep a patient register of all women obtaining an injectable contraceptive and, if the client agreed to be contacted later for a survey, to record her contact information. After following up with providers periodically, 127 providers had recorded information for injectables clients in their registers.^2^ Of these, 60 providers were later selected for in-depth interviews, of which 57 were completed (see below).

Of the 1423 users of injectable contraceptives recorded in submitted registers, 1179 had purchased DMPA-SC, and 994 of these consented to be called for a phone survey. Of 541 women completing the initial survey, 342 completed a follow-up survey timed for after they were due for reinjection. In addition, 80 women agreeing to an in-person interview in the phone survey were selected for individual in-depth interviews. After an additional 7 women were referred to the research team for in-depth interviews, 42 interviews were completed.

### 2.4 Phone survey

All consenting DMPA-SC clients (*n*=944) were contacted by phone from March to September 2016, reconsented verbally and administered a 15–20-min questionnaire by a trained interviewer bilingual in English and Yoruba. Each potential respondent was contacted up to five times; 541 women completed the first survey, while 374 were unreachable, 33 refused to participate, 22 were not eligible, and 24 phone numbers were incorrect. About half (*n*=266; 49.6%) were contacted within 1 month of their injection, 32.6% (*n*=175) were contacted within 2 months, 16.0% (*n*=86) within 3 months, and 1.7% (*n*=9) within 4–5 months.^3^ About 3 months later, timed for after respondents were due for a reinjection, all 541 respondents who verbally consented to be contacted again during the initial phone survey were called to complete a second phone survey about care-seeking for a subsequent dose of DMPA-SC, which lasted 5–10 min. In total, 342 women (63.2%) completed the follow-up survey at varying times (range 2.2–5.6 months; median 4.7 months).

From the phone survey data, we descriptively examined our sample of 541 DMPA-SC users' sociodemographic backgrounds. To assess the representativeness of our convenience sample, we used two approaches. First, we compared the sample characteristics to those of modern contraceptive users and users of longer-acting methods (i.e., injections, IUD, sterilization, and implants)^4^ in the same seven South West states from the 2013 Nigeria Demographic and Health Survey (NDHS) [4]. Differences in characteristics between our survey respondents who had switched to DMPA-SC from longer-acting methods (i.e., injectables, implants, IUDs) and the NDHS sample of contraceptive users also using longer-acting methods may provide an indication of the direction and magnitude of response biases due to our nonrandom sample. Second, to assess nonresponse bias among younger women, we compared the percentages of phone survey respondents who received DMPA-SC from a DKT Bee and were under age 25 or unmarried to a sample of DKT Bees' customer registers listing all DMPA-SC clients served, including those who did not consent.

In addition, phone survey responses to questions about previous contraceptive use (ever and in the past 12 months), obtaining another injection of DMPA-SC and side effects (“Have you experienced any side effects with Sayana Press? If so, what side effects?”) were descriptively analyzed across all respondents and between the user segments described above. Continuation of DMPA-SC was assessed based on responses to the follow-up survey and represents women who reported obtaining another dose of DMPA-SC since the time of the first phone survey, which is examined in more depth elsewhere [16]. Side effects where only asked in the follow-up survey. Thus, analyses of continuation and side effects were restricted to the subset of women who completed the follow-up survey.

### 2.5 In-depth interviews

The phone survey recruitment also served as the sampling frame for in-depth interviews with DMPA-SC providers and users. From the 127 providers who submitted register data, we purposively sampled for a mix of provider types (health facilities, drug shops and Bees). We also intended to sample for variation in DMPA-SC client volumes. However, as most providers had low client volumes, for health facilities, pharmacies and other drug shops, we selected the 15 providers of each type with the highest volumes overall (some of which did not sell any DMPA-SC during the period of observation but had sold other injectable contraceptives). For Bees, we stratified by volume and selected a mix of those with relatively high, medium and low volumes.

In August and early September 2016, after register data collection had ended, IDIs were conducted with providers following an interview guide that covered contraceptive service provision in general and specifically for DMPA-SC, and perceptions of the product's features compared to other methods offered. A total of 57 interviews were completed.

For users, we stratified phone survey respondents from Lagos, Oyo and Ogun (the three most represented states among all survey respondents) who agreed to participate in a longer, in-person interview into three groups by age and marital status: never married, married and age <25, and married and age ≥25. All women were contacted by phone to set interview appointments. Because of nonresponse and refusals among younger users, additional users were referred to the study by Bees. Between September and October 2016, 42 user interviews were completed (at varying times from the first injection), focusing on the care-seeking experience and reasons for choosing DMPA-SC as a method.

IDIs lasted 30–60 min and were conducted in English or Yoruba with a bilingual, trained interviewer following a semistructured interview guide. Interviews were translated as needed, transcribed and analyzed thematically in Dedoose using an open-coding approach in which codes were derived from the data. The codebook was continually revised as new interviews were coded; the analysis process indicated that data saturation was reached for both groups, as few codes were added toward the end of the coding. We focus here on code families that were related to the decision to use DMPA-SC and reasons for its (dis)continuation among users, along with interactions with the Bees. We focus on codes related to Bees' marketing strategies and challenges faced with the program model, as well as providers' views on DMPA-SC, for the provider interviews.

### 2.6 Ethical approvals

The Institutional Review Board at the University of California, San Francisco (IRB# 15-18353) and the Nigeria Health Research Ethics Committee (NHREC/01/01/2007-06/01/2016) approved this research.

## 3 Results

### 3.1 Product distribution

**Fig. 2:**
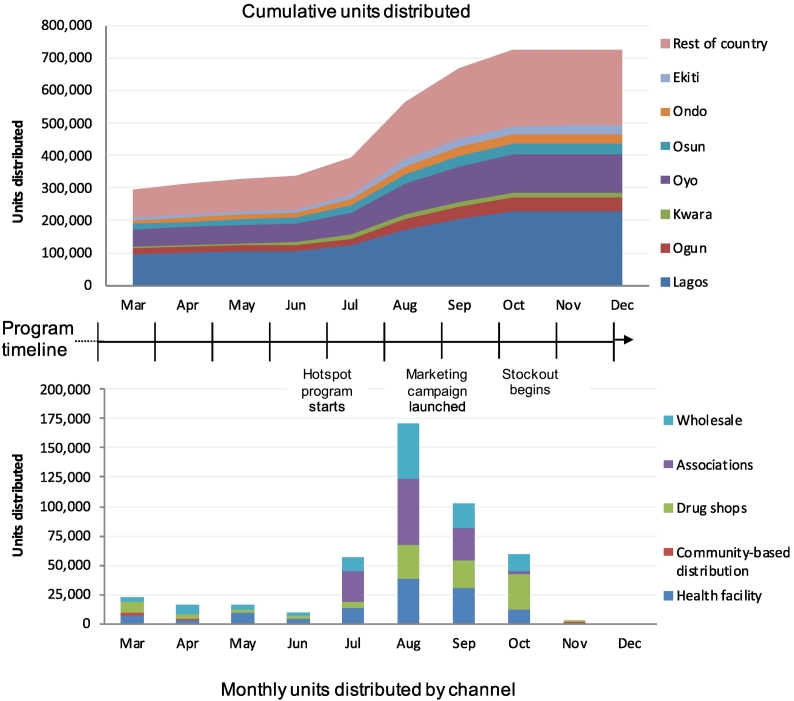
DMPA-SC units distributed through December 2016, cumulative and by month. Distribution began slowly during the first half of 2016, during which health facilities and drug shops constituted the large majority of sales (64%). Distribution rapidly increased beginning in August 2016, coinciding with procurement of a new stock of DMPA-SC with a longer expiration, a pivot toward focusing on high-volume contraception providers through the “hotspot” program, and a larger share of distribution through wholesale distributors and institutions (together accounting for 57% of all sales in Quarter 3 of 2016). During the final months of 2016, sales were severely hampered by a product manufacturing delay. Stocks for distribution were depleted in November 2016, and a reversal of the sales momentum was observed; through the end of 2016, a cumulative total of 726,675 units were distributed.

DKT Nigeria's distribution of DMPA-SC began slowly in the first half of 2016, delivering approximately 300,000 units through March (see Fig. 2 for distribution over time). During this period, DKT Nigeria prioritized distribution to direct service provider channels: health facilities and retail drug shops accounted for 64% of DMPA-SC sales. By August of 2016, several factors contributed to a rapid increase in distribution volume. First, a new stock of extended shelf-life DMPA-SC (through 2020; previous stock was set to expire in 2016) became available, which provided a foundation for greater market uptake. Second, the “hotspot” program began, facilitating the purchase of large volumes of DMPA-SC through the tiered reward system. Third, there was an increase in distribution through two supply channels: (1) wholesale, whose distributors may have been more confident about stocking larger volumes of DMPA-SC due to the longer expiration and emerging demand, and (2) professional associations (e.g., doctors, nurses, and pharmacists), with whom DKT Nigeria had made a concerted effort to connect with at national, state, zonal and local chapter meetings. Together, wholesale channels and sales to associations accounted for 57% of all sales in Quarter 3, 2016. Coinciding with the beginning of the hotspot program, the percentage of facilities placing reorders each month also increased substantially, from an average of 14.2% in the first half of 2016 to 34.3% from July to November 2016. During the final months of 2016, sales were severely hampered by a product manufacturing delay. Stocks for distribution were depleted in November 2016, and a reversal of the sales momentum occurred. Through the end of 2016, the program distributed a cumulative total of 726,675 units. Throughout the M&E period, sales were strongest in Lagos and Oyo, South West states with large urban populations.

### 3.2 Provider training and CBD model

By December 2016, 5284 individual health providers had been trained, comprised of near equal proportions of providers from retail drug shops and those based at hospitals and clinics. The distribution of trained providers across the seven focal states matched the proportional geographic distribution of DMPA-SC.

Through November 2016, DKT Nigeria also hired, trained and deployed a cumulative 211 Bees, which exceeded the KPI target for CBD workers to be trained. However, units distributed through this channel consistently fell short of milestones, only achieving a small fraction of the KPI target. High turnover of Bees also occurred during the early months, much of which was attributed either to dismissal of poor performers or to Bees disengaging due to either ineffectiveness or disinterest in proactive consumer engagement according to individual communications with staff. After program restructuring in late 2015 (with the addition of supportive supervision and more rigorous screening and training processes), the Bee program began to deliver consistent month-to-month increases in units administered by early 2016, reaching a peak of 7% of all units cumulatively distributed; these gains were temporary, and sales through CBD plateaued and stagnated. Despite further adjustments to the compensation structure, the Bee program's contribution to overall DKT sales numbers remained low throughout the remainder of the project period, accounting for <1% of all units distributed.

### 3.3 DMPA-SC users' profiles

Although Bees contributed only a small portion of DMPA-SC sales, they comprised the majority of providers (66.5%, *n*=356) from whom respondents captured in the phone survey obtained DMPA-SC; only 33.5% (*n*=179) reported obtaining DMPA-SC from a private clinic, retail drug outlet or government hospital.^5^ The overrepresentation of Bees' clients in the survey data was due to greater compliance with register keeping among the Bees as compared to other provider types. The sociodemographic characteristics of phone survey respondents reached in the initial round are summarized in Table 1. Over 80% of respondents were aged 25 or older (*n*=469) and married (*n*=503), and primarily from the top two wealth quintiles (*n*=475). Across the four user segments, 35.5% (*n*=192) were new users of modern contraception, 35.3% (*n*=191) were current users switching from noninjectables, and 29.0% (*n*=157) were switching from other injectables (e.g., DMPA-intramuscular or Depo Provera, Noristerat); only 10.0% (*n*=54) were young or unmarried. A comparison of background sociodemographic characteristics across provider types showed that respondents who received DMPA-SC from Bees were not more likely to be from poorer, younger or lower-educated groups (data not shown).

**Table 1:** DMPA-SC phone survey user characteristics compared to NDHS 2013

	Phone survey of DMPA-SC users				NDHS 2013 sample from urban areas in 7 South West states^a^	
	Initial round full sample		Switched from longer-acting methods^b^		All modern methods	Longer-acting methods^b^
	*n*=541	%	*n*=171	%	%	%
Age						
<25	47	8.7%	6	3.7%	23.4%	4.7%
25–34	287	53.0%	80	49.7%	33.9%	27.7%
35+	182	33.6%	75	46.6%	42.7%	67.7%
Marital status						
Not currently married	33	6.1%	6	3.6%	31.6%	10.1%
Currently married	503	93.0%	161	96.4%	68.4%	89.9%
Education						
Primary or less	78	14.4%	24	14.2%	26.5%	41.3%
Secondary	310	57.3%	94	55.6%	50.2%	41.8%
College/university	148	27.4%	51	30.2%	23.3%	16.9%
Religion						
Muslim	368	68.0%	57	33.3%	61.8%	58.9%
Christian	170	31.4%	113	66.1%	36.9%	39.3%
Parity						
0–1 child	76	14.0%	10	6.1%	32.2%	4.8%
2–3 children	255	47.1%	84	51.2%	25.6%	26.6%
4+ children	181	33.5%	70	42.7%	42.2%	68.6%
Wealth quintile						
Poorest, poor, medium wealth	66	12.2%	22	12.9%	12.0%	16.0%
Wealthy	109	20.1%	33	19.3%	28.2%	31.7%
Wealthiest	366	67.7%	116	67.8%	59.8%	52.3%
Place of purchase						
Private hospital/clinic/provider	65	12.0%	19	11.1%	21.1%	35.5%
Retail drug outlet	51	9.4%	17	9.9%	47.6%	6.2%
DKT Bee	356	65.8%	131	76.6%	0.64%	0.1%
Government hospital/clinic	63	11.6%	4	2.3%	51.8%	55.2%
State						
Ekiti	41	7.6%	4	2.3%	5.1%	4.4%
Kwara	15	2.8%	3	1.8%	8.3%	8.6%
Lagos	163	30.1%	59	34.5%	30.8%	18.5%
Ogun	128	23.7%	26	15.2%	10.8%	14.1%
Ondo	45	8.3%	15	8.8%	10.6%	11.6%
Osun	36	6.7%	10	5.9%	13.2%	12.4%
Oyo	111	20.5%	54	31.6%	21.4%	30.4%
Prior contraceptive use						
None (new users)	154	28.5%	0	0.0%	N/A	N/A
Switched from noninjectable	191	35.3%	14	8.2%	N/A	N/A
Switched from other injectable	157	29.0%	157	91.8%	N/A	N/A
Continuing user, but no method used in past 12 months	39	7.2%	0	0.0%%	N/A	N/A

N/A = not applicable.

a Adjusted for sample weights.

b Longer-acting methods include injections, implants, IUDs and sterilization.

To assess sample representativeness, we found that women switching to DMPA-SC from longer-acting methods captured within the phone survey and urban women residing in the south west using the same contraceptive methods sampled in the 2013 NDHS were similarly overwhelmingly older and married, but phone survey respondents were more educated (*n*=24/171, 14.2%, had primary schooling or less) compared to NDHS sample (41.3%).^6^ A comparison of the age and marital status for the subset of phone survey respondents who attended DKT Bees to a sample of complete DKT Bees' customer registers indicated that the percentages of users under age 25 and who were unmarried were not substantively different (data not shown).

### 3.4 User experience with DMPA-SC: Method choice

In user IDIs, the most commonly reported reasons for why users chose DMPA-SC as their preferred method of contraception were (1) trust in provider recommendations, (2) recommendations from friends and (3) appreciation for DMPA-SC's 3-month duration of effectiveness. The 3-month duration was attractive both to women who wished to resume childbearing in the near future and to those who had either previously been using shorter-duration methods or who had become accustomed to DMPA-intramuscular's 3-month duration.

*[My provider] told me she has heard about Sayana Press, that it works that, and that I should just try it. I said okay, let me just give it a try.*

Lagos, age 22, never married, source from a DKT Bee, current user switching to DMPA-SC.

*I have a friend that got Sayana Press and I made up my mind because she said it was okay.*

Lagos, age 32, married, source from a DKT Bee, new user

Among the subset of DMPA-SC users who were switching from another method of modern contraception, the most commonly mentioned reasons for choosing DMPA-SC were (1) reduced side effects with DMPA-SC as compared to the other method, (2) trusting the recommendation of a friend and (3) appreciation for DMPA-SC's 3-month duration of effectiveness.

*I was told that the milligram of Depo is more than Sayana Press…and I was told that though Sayana can cause the same effects as Depo, they aren't as much as with Depo.*

Oyo, age 30, married, source from a DKT Bee, current user switching to DMPA-SC.

Less frequently mentioned reasons for switching to DMPA-SC included (1) the user's usual provider ceased offering non-DMPA-SC injectables, (2) trust in provider recommendation and (3) wanting to try DMPA-SC in hopes of experiencing fewer side effects than other methods, but particularly Depo Provera.

*I went there for Depo injection, but they said there was no Depo, the person that was on duty, I [trusted her], and she advertised the new product to me, so it is not that I wanted them to give me Sayana Press, but because of my belief in them that I changed to Sayana Press.*

Oyo, age 22, married, source from private clinic/hospital.

*When I told her that my [old method] normally gives me headache, she says I should leave that one and come and be taking Sayana Press.*

Lagos, age 37, married, source from private clinic/hospital.

### 3.5 User experience with DMPA-SC: satisfaction and continuation

In user IDIs, among both new users of modern contraception and current users switching to DMPA-SC, positive experiences with DMPA-SC were generally associated with having no or minimal side effects, satisfaction with the effectiveness of the product and the resulting peace of mind. About half of respondents followed up at after 3 months reported experiencing a side effect (53.2%, *n*=182/342). Across user segments, the absence of reported side effects was highest among young and unmarried users (61.5%, *n*=16/26), followed by current users switching from other injectables (57.3%, *n*=59/103) and noninjectables (45.2%, *n*=52/115), and lowest among new users (41.2%, *n*=55/128). From IDIs, the most frequently mentioned side effects pertained to changes in menstruation, followed by changes in weight.

*What I don't like is that since I've collected the second dose, the flow of my menstruation has been much. There was nothing that happened when I used the first dose, my menses was normal but the second one was a problem to me, till now it has not stopped.*

Lagos, age 28, married, source from a DKT Bee, new user.

According to the follow-up phone survey round, 50.6% (*n*=175/346) of respondents had obtained another injection of DMPA-SC; current users switching from other methods were the most likely to continue between phone survey rounds (53.4%, *n*=55/103 among those switching from another injectable; 53.0%, *n*=61/115 among those switching from a noninjectable); younger and unmarried users were the least likely to continue (38.6%, *n*=10/26). Across all user segments, the most common reasons given for discontinuation were related to side effects (21.8%, *n*=22/101), though a nontrivial percentage of women reported that they intended to become pregnant again (10.9%, *n*=11/101). Nearly all women who obtained a second dose did so from the same provider from whom they received the first dose (96.6%, *n*=169/175). In IDIs, users further reported that being counseled about the possibility of side effects when receiving the first dose of DMPA-SC and provider availability for follow-up if side effects were experienced were both helpful to either manage or tolerate side effects while also encouraging continuation.

*…I now explained to her that I saw my menstruation, she now said “it doesn't matter,” that it doesn't do anything, that it is just giving me sign that that injection has started working in my body, that I should not be afraid.*

Ogun, age 24, married, source from a CBD, new user.

### 3.6 Provider views on DMPA-SC

Across the different provider cadres participating in IDIs, respondents had generally favorable views of DMPA-SC. They positively noted the ease of administering the product and its small needle, although some also mentioned that it was more expensive than other contraceptive methods, which was a barrier for their clients. As with DMPA-SC users, side effects were a main consideration in providers' views of DMPA-SC. Most said that they received relatively few complaints about side effects from clients using DMPA-SC, and several specifically compared this favorably to clients' experiences with DMPA-intramuscular.

*The advantage is because it contains [a] lower [dose of] prostegerone it reduced [the] side effect of Depo Provera and we have fewer complaint from the people that are using Sayana Press.*

Clinic, Lagos.

However, providers also noted that some clients still experienced side effects with DMPA-SC, particularly changes in menstruation, which along with cost were the main disadvantage they saw in the product.

### 3.7 Provider and user experience with the CBD model

Because the majority of phone survey and user IDI respondents obtained DMPA-SC from a Bee, more information on users' experiences with this cadre emerged than from other providers. Nearly all respondents reported high satisfaction with their encounter with the Bee. Compared to other providers, a higher percentage of Bees were reported to have asked the user if she had experienced side effects before (87.1%, *n*=310/356 vs. 80.4%, *n*=148/184) and conducted a pregnancy test during the counseling session (84.8%, *n*=302/356 vs. 77.7%, *n*=143/184) among phone survey respondents. In the IDIs, users reported positive experiences with Bees who came door-to-door to meet them and advertise their products, including DMPA-SC. Approachability and trustworthiness were key facilitators of user receptivity to the Bees. Users were also responsive to the strategy of offering the first dose for free, while others reported that they appreciated that Bees reminded them when it was time for their next dose of DMPA-SC.

*I was at my auntie's shop when the DKT Bee approached me. She discussed Sayana Press with me, gave me handbill to read, and gave me her phone number that day. Then she said I should call her if I want to use it. I called her after some time to say that I wanted to use Sayana Press.*

Oyo, age 23, never married, new user.

*The DKT Bee came to advertise to me and when I see she is a good person and not someone that will be telling me lies that was why [I decided to take DMPA-SC from] the DKT Bee*

Ogun, age 24, married, unknown user status.

Bees themselves similarly described the personal relationships and more effective strategies for proactively engaging women in community-based settings that facilitated their work. Most Bees reported success in recruiting DMPA-SC clients by (1) going house-to-house and engaging with clients in the privacy of their homes; (2) meeting with women in large groups, in venues such as markets, churches or at gatherings of trade associations; and (3) getting referrals from other clients.

*I go from house to house. I introduce myself and then after I make the familiarities from house to house, [I also go] to the market and even to the church, so as to create awareness both for males — the husbands — and the women, and they are responding positively.*

Ogun, DKT Bee.

However, Bees also mentioned numerous challenges of their job, key among which were (1) difficulties in overcoming misinformation and negative community attitudes toward contraception, (2) high transportation costs and difficulty covering large geographic catchment areas and (3) selling products on credit because clients could not afford them (including DMPA-SC).

*To enlighten, to convince [women], it is not easy. A lot of women are totally ignorant [about contraception], and we have to start from scratch with them.*

Lagos, DKT Bee.

*The challenge is that people will tell you there is no money, and for that I sell on credit. And some will even say that their [next dose] is due, and after collecting injection, they will tell you there is no money.*

Ogun, DKT Bee.

These challenges likely contributed to the Bees' lower-than-expected volumes of sales, which also affected their financial incentives to continue with the program.

## 4 Discussion

Although the private sector introduction of DMPA-SC in Nigeria encountered a number of challenges, DKT Nigeria's course corrections and problem-solving highlighted a number of strategies that can inform future DMPA-SC programming. Information from the phone survey and in-depth interviews provided initial evidence that both users and providers viewed their experiences with DMPA-SC favorably. Although perspectives were only captured from a few younger women, comparison of the phone survey sample with the representative NDHS suggests that women choosing longer-acting methods are older in general and that the small proportions of younger women captured in the M&E may provide a reasonable reflection of program reach rather than resulting wholly from sampling biases. Thus, the sizable proportions of new users of contraception and current users switching from noninjectable methods (about one third of respondents for each category) surveyed suggest that the addition of DMPA-SC into the method mix may be expanding the market for contraceptives in Nigeria.

Based on our mixed-methods M&E of the private sector introduction of DMPA-SC in South West Nigeria, our findings can be synthesized into four main lessons regarding contraceptive social marketing in Nigeria.

*Lesson 1: Targeting high-volume contraceptive service providers and leveraging existing wholesale distribution channels helped to catalyze product distribution.*

Strategies deployed by DKT to increase distribution highlight the advantages of the social marketing approach through commercial markets, including targeting high-volume contraceptive service providers and leveraging wholesale distributors. Distribution of DMPA-SC accelerated toward the end of the program due not only to the availability of new stocks of DMPA-SC with longer shelf life but also because of an increased focus on improving efficiency in the supply chain: the shift away from engaging with a diffuse array of individual providers and toward the “hotspot” approach of targeting high-volume contraceptive service providers, all while simultaneously increasing wholesale distribution. Both strategies were founded in the social marketing approach of leveraging existing market and supply chain structures. Targeting high-volume providers and wholesale distribution channels further ensured that the product was distributed to service delivery points that were likely to have established demand among the consumer base. While direct data on actual use were lacking and it is therefore difficult to generate direct evidence on the size of the market for DMPA-SC, the percentage of facilities reordering DMPA-SC also climbed after the hotspot program began, suggesting that use of DMPA-SC was increasing.

*Lesson 2: Community-based distribution was well received among DMPA-SC users, but the Bee program struggled with balancing cost recovery for sustainability and the public health goals of reaching underserved populations.*

In general, CBD models hold promise for encouraging uptake of modern contraception, particularly among women who are unlikely to seek contraceptive services from existing health facilities and providers [17], [18], [19]. The DKT Bee program sought to deliver DMPA-SC in large quantities to women, particularly to women in underserved communities. Despite efforts to optimize operations, the program was unable to achieve its service delivery goals. Primary challenges included recruiting and retaining CBD workers, establishing suitable compensation structures and sales targets while supporting logistical costs, and providing supportive supervision for proactive community-based work. Unique to the Nigerian context, the CBD scope of work did not align with those of the licensed CHEWs recruited for the program. Although not originally intended, Nigerian CHEWs have come to expect to deliver facility-based services rather than through CBD [14]. As a result, the CBD model proved a difficult adaptation for many despite recent government task-shifting policy that empowered CHEWs to deliver injections and address gaps in service provision [13].

Even though women reached through CBD were overrepresented in the data collection relative to the channel's share of overall sales, results from the phone survey showed that respondents who received DMPA-SC from Bees were not more likely to be from poorer, younger or lower-educated groups. This suggests that Bees were not able to disproportionately reach underserved populations. A key challenge in this regard may have been the cost of DMPA-SC relative to other contraceptive products. This experience with CBD in Nigeria's private sector highlights the difficulty of meeting goals associated with cost recovery, primarily through quantity of sales, while also seeking to achieve public health priorities for serving marginalized populations, which are often more difficult to reach and have lower ability to pay. These potentially diverging objectives may require different programmatic strategies, particularly if delivered through the private sector; cost recovery may be limited if strong motivational incentives are needed for last-mile service provision. To overcome affordability barriers for underserved women, targeted subsidies may further help achieve utilization goals while maintaining the retail price for the overall market.

Despite the program's challenges, interview responses from users served by Bees indicated that the CBD approach was an important factor in encouraging some of them to take up contraception and DMPA-SC, which suggests that service delivery through CBD does have a place in the contraceptive health market. Several strategies to lower program costs and generate more revenue could be considered for future private sector CBD programs in Nigeria. These include (1) screening for more entrepreneurial individuals and providing supportive supervision [20], which helped to improve worker performance to some extent among the DKT Bees: (2) introducing competitive compensation structures to incentivize more home visits [17]; and (3) expanding the range of products offered beyond contraception. Notably, among the few Bees who remained active throughout the program, the marketing strategies they employed included both house-to-house visits and organizing group sessions at different community locations and events (markets, churches, trade association meetings). Greater integration of community health workers with facility-based providers may help to forge mutually reinforcing relationships for client referrals and continuity of care [20].

*Lesson 3: Recommendation from providers and family/friends, along with the quality of counseling, can influence the decision to start contraception or switch to DMPA-SC from another method.*

Given the restrictions on direct-to-consumer marketing of DMPA-SC, DMPA-SC brand or product awareness among consumers was likely dependent on word-of-mouth or on providers' initiative in introducing DMPA-SC as part of the contraceptive method mix. Indeed, survey respondents most commonly mentioned that trust in a provider's or a family member/friend's recommendation was the primary reason they chose to use DMPA-SC. Despite the challenge of creating specific awareness for DMPA-SC among consumers more broadly, the percentage of new users of modern contraception captured in our phone survey fell within the range documented in other DMPA-SC pilot countries [21]. The substantial portion of respondents who were new users suggests that introducing DMPA-SC into the method mix may be expanding the market reach for contraceptive services in Nigeria. Among women switching to DMPA-SC from another method, many viewed DMPA-SC as a direct replacement for DMPA-intramuscular but were attracted to DMPA-SC because they heard about the potential for decreased side effects.

The quality of user interactions with DMPA-SC providers and user experience with side effects were significant contributors both to women's positive experiences with DMPA-SC and to user decisions to continue with DMPA-SC. In a companion paper, we found that the quality of counseling and the experience of side effects both independently influenced users' reported continued use of DMPA-SC after 3 months [16]. User interviews also suggested that when women were briefed on potential side effects by providers, they were more likely to report that side effects were manageable and more likely to have favorable feelings about DMPA-SC even if they experienced side effects. Similar to studies on quality of contraceptive counseling in other settings [22], [23], interpersonal communications and providers' ability to give clients accurate information about potential side effects, along with strategies for managing those side effects, were particularly important. Given the strong influence of providers on women's contraceptive decision making, provider training will remain vital for helping them to deliver a high-quality contraceptive service interaction.

*Lesson 4: Targeted efforts are needed to reach younger, unmarried users.*

The DMPA-SC program in Nigeria struggled to reach younger and unmarried users despite explicit efforts such as the DKT Bee delivery model to do so, echoing the larger inequities in health and access to services for these groups [24], [25], [26]. Younger and unmarried users comprised a small percentage of women not only in the user phone survey and in-depth interviews but also within Bees' complete customer registers and among longer-acting method users sampled in the 2013 NDHS, mirroring existing programmatic challenges in reaching these segments with contraceptive services. With future DMPA-SC programming, it will be important to differentiate (1) a mass marketing approach for DMPA-SC aimed at generating and meeting demand from a traditional Nigerian contraceptive consumer base from (2) tailored approaches for specific populations of interest, particularly young and unmarried women (along with new users, women in rural areas and low-income families). Careful consideration should be given to the channels through which young and unmarried women access the existing health system, particularly for sexual and reproductive health. Many providers maintain biases against the provision of contraception to younger and unmarried women [27], [28], [29]. Thus, several strategies may be more effective for reaching this group: recruiting young women to serve as program-affiliated community education and advocacy staff to supplement CBD, screening out providers with biases and/or engaging providers with tactics for holistic youth-friendly service provision [30].

### 4.1 Limitations

A number of challenges for data collection were encountered as a result of studying a market-based program under real-world conditions. Most program data could not be independently verified, and data systems were not able to capture actual product administration for non-CBD providers. No registration database or standardized reporting formats exist for providers in Nigeria's private sector, even for facilities. Thus, representative samples could not be readily drawn from the population of providers or patients. The resulting samples of providers supplied by the distributor and who consented to participate in the M&E, and subsequently the sample of women responding to the phone survey and IDIs, were skewed not only toward those in urban areas (i.e., Oyo and Lagos states) where DMPA-SC distribution was more concentrated but also toward Bees who were more likely to comply with information requests by virtue of being directly supervised and employed by the implementer. Although enumerators followed processes to validate and verify the accuracy of information collected in patient registers given to providers, it is unknown to what extent providers recorded information for all eligible women they attended to; many young or unmarried women may have also declined to have their information recorded. In addition, many women could not be reached via phone, while a smaller number declined to participate due to confidentiality reasons — problems particularly challenging among young or unmarried users. All data collection activities excluded women without access to a telephone, who are likely to be poorer and from more rural locations.

As such, quantitative survey results are unlikely to be representative, and caution should be used in interpreting these results. In particular, responses overwhelmingly reflect women attending Bees, who only comprised a small number of DMPA-SC providers and represent a specialized cadre mobilized only for this effort. Therefore, the experiences of these women may not reflect the more typical experiences of women seeking contraceptive services from established private providers. Although imperfect, our comparison of sociodemographic characteristics between phone survey respondents and the NDHS was one attempt to assess the extent of these biases; the similarities in sociodemographic characteristics between women using longer-acting, reversible methods in both samples suggest that the phone survey may have at least captured a segment that may be somewhat generalizable to the larger population. In addition, respondents, and particularly Bees, may have felt pressured to report favorable responses in interviews by virtue of the association of the research activities with DKT Nigeria. While research conducted in a more controlled environment may have yielded more robust, internally valid inferences, it would have similarly lacked external generalizability and would be less well suited to studying real-world programs that are quickly iterating and scaling. A more controlled approach would also have inhibited providing timely feedback to facilitate program course correction; the research design adopted, with its limitations, is also an important lesson learned in terms of the challenges of conducting M&E for a mass market, private-sector-based program.

## 5 Conclusions

Data collection challenges notwithstanding, the DMPA-SC private sector introductory program in Nigeria has substantially evolved over time, generating a number of important lessons learned for future market-based introductions of a new contraceptive product. Distinguishing between broad-based and targeted outreach strategies for different groups of potential users at different stages of a method introduction is critical for such programs, including clearly defining the goals and role of CBD. The undertaking was also instructive for understanding how continuous M&E could help to document challenges and successes on an ongoing basis. Understanding the experiences of different key user market segments in obtaining and using DMPA-SC across different channels was helpful for assessing program reach and identifying areas for program improvement, particularly for what women valued in their provider interactions and the challenges faced in reaching young and unmarried women.

## Conflicts of interest

The authors declare no conflict of interest.

## Funding

This work was supported by the Bill & Melinda Gates Foundation, Seattle, WA (grant number OPP1133271) and the Children's Investment Fund Foundation, London, UK (grant number N/A). Program officers from these Foundations contributed to the overall objectives of the research portfolio and provided feedback on research protocols and instruments. However, the authors had sole responsibility and authority for the collection, analysis and interpretation of data, the writing of the report, and in the decision to submit this article for publication.

## Appendix A Supplementary data

Supplementary material Image 1

## Footnotes

1 CHEWs are a special cadre of health workers in Nigeria employed in the public sector who undergo formal training and are permitted to administer injectables [13].

2 Reasons for providers not supplying any client information included electing not to fill registers, losing registers, forgetting to do so and closure of the business.

3 An additional five women did not respond to the question of how long ago they received their injection.

4 It was not feasible to restrict the NDHS sample to only injectable contraceptive users as the resulting sample size was too small to yield valid population-level inferences.

5 During provider recruitment, it was discovered that some providers purchasing DMPA-SC were public facilities inadvertently misclassified as a private facility.

6 Because the phone survey sample of users were more comparable to the 2013 NDHS sample of modern contraceptive users residing in urban areas rather than rural areas, we only show the profile comparison to the NDHS urban sample.
