# Supplementary material for: Introducing the subcutaneous depot medroxyprogesterone acetate injectable contraceptive via social marketing: lessons learned from Nigeria's private sector
Source: Contraception. 2018 Nov;98(5):438–48. doi: 10.1016/j.contraception.2018.07.005 (PMC6197840; doi:10.1016/j.contraception.2018.07.005)
Supplement: Supplementary file 1 — Supplementary material [file mmc1.docx]

**Supplementary materials**

**A. DKT Nigeria and the DMPA-SC introduction program**

DKT is a non-profit organization that promotes family planning and HIV/AIDS prevention through social marketing. In 2014, DKT established an office in Nigeria, based out of Lagos. DKT Nigeria aims to increase contraceptive usage and availability in Nigeria. The concept of contraceptive social marketing involves leveraging existing market infrastructure, incentives, and methodologies to ensure that men and women across Nigeria have access to accurate knowledge about contraception, quality products and services (for more information, please see <http://dktnigeria.org/>). The DMPA-SC program included medical detailing to providers, distribution to outlets and facilities, marketing campaigns on multi-media platforms, and leveraging mobile technology to provide a reminder service that encourages method continuity.

*Product sales*

As of 2018, DKT Nigeria distributed a basket of products which include condoms (brands Fiesta and Kiss), daily contraceptive pills (Levofem), emergency contraception (Postpill), implants (Implanon NXT, Jadelle), various IUDs (Lydia, Eloira), the DMPA-SC contraceptive injection (Sayana Press), misoprostol and mifepristone (Misofem, Mifepak), manual vacuum aspiration kits, and maternity kits. DKT Nigeria leverages existing commercial and market channels and networks for operational efficiency and scalability. DKT Nigeria's primary clients are drug shops, hospitals and clinics, supermarkets, and wholesalers. DKT Nigeria uses a dedicated salesforce deployed to different regions to distribute products to providers. Supervised by experienced Sales Managers, a team of Medical Sales Representatives perform medical detailing, identify training needs, and ensure product availability in their territories. Sales teams carry and distribute the full range of contraceptive products, organize product presentations, and circulate promotional and education materials to both providers and clients. DKT Nigeria also guaranteed delivery of products to providers within 12 hours to ensure timely provision of quality-assured contraceptive and safe abortion services.

DKT Nigeria also has relationships with wholesalers to achieve greater depth and breadth of distribution to the medical services retail sector. Wholesalers, who can expand distribution coverage to smaller facilities (especially outlets with lower purchasing capacity), complement DKT Nigeria’s own sales staff and enable DKT products to be available at more outlets throughout the country.

Lastly, DKT Nigeria has partnerships with the medical community and participates in provider association meetings and conferences to build product visibility. These include professional associations for medical doctors (OB/GYN in particular), nurses and midwives, CHEWs, pharmacists, and patent and proprietary medicine vendors. Regular meetings for these associations are held at national, state, and local levels where DKT sales representatives make presentations and sell directly to interested providers.

In October 2015, electronic data capture was instituted to track all transactions conducted by medical and sales representatives to providers. Over the first several months, the system was further refined and data entry improved for consistency and quality. However, this system was not able to track provision of products, including DMPA-SC, from providers to clients. Thus, the distribution data presented for key performance indicators (KPIs) is a proxy for product uptake. This database was used to optimize distribution, giving field personnel and managers real-time information into sales activities, distribution routes, provider information, and gaps in services and products.

*The DMPA-SC introduction program in Nigeria*

In 2015, DMPA-SC was first introduced in Nigeria with a focus on the proximal south western states (Lagos, Oyo, Ogun, Osun, Ondo, Kwara and Ekiti). The product was integrated into distribution and medical detailing activities of the salesforce and via CBD using licensed CHEWs, and later combined with marketing campaigns to drive general awareness about contraception and DMPA-SC in particular. DMPA-SC was sold to providers at 250 Naira (~US$1.25) per injection, which was about 100-150 Naira more than for Depo Provera or Noristerat, the other injectable contraceptives available in Nigeria at the time. The recommended retail price was 500 Naira (~US$2.50) per injection.

The DKT Nigeria DMPA-SC program included two features that were specific to the product. First, a mobile SMS text message service, started in November 2015, enabled subscribers to receive automated reminders timed for their next injection. Notification of this service was printed on small labels and affixed to the exterior of individual DMPA-SC packets; separate small leaflets were also printed. Providers were instructed to explain the reminder service to their clients and help them enroll in the service if interested.

Second, a proactive CBD system through licensed CHEWs was created, called the DKT Bee program. CHEWs are a cadre of health workers in Nigeria that undergo 3 years of formal training and are tasked with the provision of many basic primary care services, including the provision of injectable contraceptives.[1] After training, CHEWs are intended to be employed at public sector primary health centers, and are expected to provide facility-based services for most of their time and community-based services for a minority of their time.[2] In the DKT Bee program, CHEWs were directly recruited, trained, and managed by DKT Nigeria to offer contraceptive products and referrals by proactively engaging clients in community settings (for more details, see below).

Initial consignments of DMPA-SC from the manufacturer had limited a relatively short shelf life, expiring within a year of receipt and which made the product less attractive to providers. Medical and sales representatives also needed to revisit providers to replace expired stock. New DMPA-SC consignments with a longer shelf life were received in mid-2016, which facilitated several growth trajectories for distribution. First, the timing of this new consignment helped to encourage higher volume purchases from both providers and wholesale distributors, who were previously wary of carrying expired product. Second, a concerted effort to cultivate relationships with medical professional associations (e.g., doctors, nurses, midwives, CHEWs, proprietary and patent medicine vendors) was also launched. Sales conducted at meetings of different medical personnel at national, state, zonal, or local levels helped to drive greater visibility of DMPA-SC and ultimately higher distribution volumes. Third, stock with longer expiry enabled DKT Nigeria to further expand DMPA-SC distribution to seven additional states in the north (Kano, Kaduna, Kogi, Niger, Nasarawa, Abuja, and Plateau) and beyond.

*Provider training*

DKT Nigeria offers health care providers training on contraceptive service provision of the products they supply. Early in the DMPA-SC program, providers were identified through sales and medical representatives during medical detailing and invited to participate in trainings held off-site in a central location. Because irrelevant personnel who were not offering contraceptive services or had no intentions to do so were attending these training events, the format was altered. Training sessions were instead held on-site at the facility and all relevant personnel (i.e., doctors, midwives, nurses, CHEWs, and auxiliary nurses) at the facility were invited to attend. Facility-based training helped to ensure that only providers interested in offering contraceptives services and who bought DKT Nigeria’s quality-assured products were trained, improving the targeting, effectiveness, and efficiency of training. In addition, the facility-based training format also helped to ensure that, at any given facility, at least one health worker was capable of offering contraceptive services and fill gaps due to health worker turnover.

Training for providers was carried out using the PATH training curriculum for DMPA-SC.[3,4] Training modules included a general introduction to contraceptive methods and counseling techniques, as well as specific modules on the DMPA-SC injectable, its unique features, benefits and administration, side effects management, and the availability of the free reminder alert service for clients (see below). Trainings also included injection practice sessions on simulation dummies to ensure competency with the procedure, and pre- and post-tests were given to assess learning and comprehension. Training sessions lasted three to four hours, led by Master Trainers who were licensed and practicing OB/GYNs doctors, nurses, and community pharmacists. DKT employed nurses to train CHEWs and other lower level cadres (e.g., drug shop staff and owners). Each participant was given a certificate of attendance.

*The DKT Bee community-based distribution program*

The DKT Bees Program was developed as a channel for offering personalized contraceptive services directly to women in their home or business, at markets, and other community-based settings. The program was initially conceived of as filling a service gap for lower-income and marginalized populations that face barriers to seeking care at health facilities. Licensed CHEWs were recruited and trained to be a “DKT Bee,” outfitted with specially-branded uniforms, cooler bags, and contraceptive products. In addition to selling and administering DMPA-SC, Bees sold a basket of contraceptive products, including condoms and oral contraceptive pills. They were also equipped with pregnancy tests and many carried blood pressure cuffs. Initially supplied with products for free, Bees were expected to purchase additional replacement stock. Markups on the retail price of any products sold then comprised their income with bonuses given for reaching set volume thresholds. Their performance was measured based on the number of contraceptives sold, including DMPA-SC units administered to women.

Although DKT Bees were recruited and deployed in all seven introduction states at one time, initial turnover was high and retention poor, gradually leading to DKT Bees only being active in urban areas in Lagos and select other cities in the South West. According to discussions with program staff and Bee supervisors, many Bees did not like the sales approach to the program because they were expecting women to seek care from them at a facility or primary health center. Many Bees dropped out of the program because CBD was not a practice common in their CHEW training.[2]

To address retention challenges, the program was occasionally adjusted to attract, recruit, and retain entrepreneurial-driven CHEWs who were more interested, motivated, and able to drive the CBD strategy. In contrast, many Bees initial dropped out of the program or were let go due to poor performance because they found CBD to be challenging and counter to their expectations of providing facility-based services common to public sector CHEW employment. In order to encourage better performance among individual Bees, DKT instituted a number of additional features into the program: (1) supervisors (i.e., “Queen Bees”), licensed nurses and selected for their outgoing and positive approach to the job, were hired to recruit, monitor, and mentor Bees, including job shadowing and on-the-job training; (2) Bees were allowed to work in pairs to boost confidence in proactively engaging women; (3) sales targets were adjusted downward based on actual performance data; and (4) the compensation structure was modified to tie Bee income directly to sales (i.e., 50% of the sale price of all items) and eliminate allowances for transportation and communications which some Bees collected without selling any products.

During the program, selected interviews with Bees indicated that better performing Bees employed several strategies for selling products: (1) relying on their own social networks for selling products; (2) approaching women in groups (e.g. religious meetings, workplaces) and providing information in more of a health talk format; and (3) selling products at a lower price than the recommended retail price or even for free for women who could not afford them.

Despite efforts to improve the program, distribution through the DKT Bees continued to fall short of anticipated targets—only 1.5% of the goal for units administered was achieved. Through attrition, only a handful of Bees continued to operate in selected geographies and the program was ultimately discontinued in late 2016.

*The Hotspot program*

Experiences during the initial months of product distribution showed that volumes for contraceptive services varied widely among providers, with many providers attending to only a few clients per month. In August 2016, DKT Nigeria embarked on a “hotspot” program which targeted relatively higher volume contraceptive providers and offered them a set of incentives (e.g. blood pressure monitors, weight scales) for large bulk product purchases. Additional support for training of all providers at the facility on contraceptive service provision (as opposed to only those involved in reproductive health) via a “whole site” approach and supplies of marketing materials (e.g., banners, flyers) were also given to hotspot providers. By December 2016, 613 facilities, pharmacies, and PPMVs were enrolled into the hotspot program.

*Marketing and communications campaign*

DMPA-SC was registered with the National Agency for Food and Drug (NAFDAC) as a prescription-only product. As such, direct-to-consumer marketing for DMPA-SC specifically was prohibited. This restriction implied that all efforts to increase client awareness would be dependent on providers’ initiative to offer DMPA-SC to clients and/or clients’ initiative to request it, which was unlikely to occur without any point of service marketing materials prompting clients to do so. These marketing restrictions were counter-productive to the objective of creating awareness of DMPA-SC, and motivated the development of a marketing and communications strategy that aimed to build general awareness around contraception and types of methods offered.

DKT Nigeria’s marketing campaign was crafted for six target audiences: (1) young, female students; (2) career women living alone; (3) illiterate married women with children in rural areas; (4) young women in the north in urban/peri-urban areas; (5) market women married with children; and (6) male partners. While no specific method was used to develop these user profiles, several types of market research methods were employed to inform specific communications strategies, including voice box recordings and photo voice. The marketing campaign utilized both traditional (radio, television) and social and online platforms, summarized in Table S1. A separate brand identity, Honey and Banana, was created to reduce emphasis and stigma associated with contraceptive products, and increase relevance to the broader consumer audience.

Table S1. DKT Nigeria marketing and communications strategies

| **Media format** | **Description** |
| --- | --- |
| Radio | - Regular programs aired in multiple languages and regions - Presentation includes a combination for entertainment and education: presenters will first engage audiences in a more entertaining scenario (e.g., young people call in and attempt to sing a song), followed by Q&A with a health worker (a doctor or nurse trained by DKT) responding to questions about health, sex, relationships, and contraception. - Segment specific to the Honey and Banana brand are produced. - Programs are tailored to the level of discreetness required for the prevailing cultural norms in broadcast areas. |
| Television | - Complete shows are filmed and aired. - Contain both educational and drama-based content (e.g. relationship and contraception-related issues) - Partnership with MTV Shuga to further increase brand recognition |
| Social media | - Active on Facebook, Instagram, Twitter, and WhatsApp - Staff field user-generated queries directly, through both public and private fora, including hosted live video chats and tweet-and-response sessions |
| Internet | - Honey and Banana website (<http://honeyandbanana.com/>) contains a variety of information about sexual health, family planning products, and answers common questions with a FAQ |

Launched in September 2016, with different activities successively launched, the full suite of media activities were in place by early 2017. Reports from media service providers suggest that online and social media platforms generated high interest among younger people. For example, individuals under age 25 constituted over 80% all users of the Honey and Banana website and that two times more women used it than men. The frequency and content of user-generated queries suggest that these digital platforms are filling gaps in reproductive health information and referrals to services.

**B. Key performance indicators**

Table S2 summarizes the key performance indicators used for the M&E of the DMPA-SC introduction in Nigeria. The objectives and associated indicators were developed in conjunction with the program funders and implementers at the beginning of the project.

| **Table S2. M&E Objectives and Indicators** | | |
| --- | --- | --- |
| **Objective** | **Indicator** | |
| 1. Track progress against key program outputs | 1.1 | Number of private providers (retail & clinical outlets) trained on DMPA-SC |
|  | 1.2 | Number of enrollments in the mobile reminder system |
|  | 1.3 | Number of units of DMPA-SC distributed (by channel, location) |
|  | 1.4 | Percentage of women sampled who were unable to find resupply |
|  | 1.5 | Percent of DMPA-SC orders each month made by previous retail purchasers of DMPA-SC |
|  | 1.6 | Percent of women sampled who encountered DMPA-SC social marketing |
|  | 1.7 | Percent of women sampled who encountered marketing for community-based distributors |
|  | 1.8 | Number of community-based distributors trained |
|  | 1.9 | Doses of DMPA-SC administered to clients by community-based distributors |
| 2. Generate a profile of DMPA-SC users, particularly uptake among new adolescent users as a result of adding DMPA-SC to the method mix | 2.1 | Client sociodemographic profile, parity |
|  | 2.2 | Percent of doses of DMPA-SC administered to women under age 20, age 20-24, and 25+ |
|  | 2.3 | Percent of sampled DMPA-SC users that are new users of modern contraception |
|  | 2.4 | Percent of sampled users that switched from other methods, by method |
| 3. Understand users’ decision to try and satisfaction with DMPA-SC | 3.1 | Perceptions of different methods compared to DMPA-SC |
|  | 3.2 | Decision-making process for method choice |
|  | 3.3 | Barriers to accessing preferred methods relative to DMPA-SC |
|  | 3.4 | Reasons for choosing provider |
|  | 3.5 | Reasons for method switching, dis/continuation |
|  | 3.6 | Concerns about family planning/DMPA-SC use |
|  | 3.7 | User satisfaction with DMPA-SC |
| 4. Compare delivery performance across different channels | 4.1 | Provider counseling quality by channel |
|  | 4.2 | Provider perceptions of DMPA-SC and potential market for product by channel |
|  | 4.3 | Reported retail amounts by channel and product (markups calculated) |
| 5. Assess key stakeholders’ perspectives on and experience with the DMPA-SC introduction | 5.1 | Perceptions of and experiences with DMPA-SC rollout |
|  | 5.2 | Perceptions of home and self-injection by stakeholders |
|  | 5.3 | Opportunities for program improvements |
|  | 5.4 | Identification of key barriers to scale-up |
|  | 5.5 | Recommendations for scale-up strategy |
|  | 5.6 | Recommendations for future stakeholder engagement |
| 6. Understand perceptions of and potential practice of self-injection among DMPA-SC users | 6.1 | Self-injection perceptions |
|  | 6.2 | Home and self-injection readiness/interest |

**C. Recruitment**

From a list of 358 providers who purchased DMPA-SC from DKT Nigeria between October 2015 and February 2016, we enrolled a convenience sample of 205 healthcare providers to act as DMPA-SC user recruitment sites. Information on providers purchasing DMPA-SC prior to October 2015 was not available as prior sales transactions were not electronically captured. Providers included hospitals, clinics, and maternity homes, which usually provide the full range of contraceptive products available in Nigeria, drug shops that sell a more limited range of contraceptive products (i.e. condoms, oral contraceptive pills, and injectables), and Bees directly trained and recruited by DKT Nigeria to provide short-acting methods and DMPA-SC as per CHEWs’ legal scope of practice.^[[1]](#footnote-1)^ The initial list of health providers referred to the M&E team was first cleaned to remove duplicate entries and entries that did not have enough identifying information to be contacted (i.e., no address or phone number). Of the remaining 316 unique providers, all were contacted first by phone in order to verify address and location information, and then visited in person by two members of the team for recruitment into the study.

M&E team members tasked with provider recruitment were trained over a four-day period, focusing on establishing positive rapport with providers in order to facilitate later follow-up. Training topics included an overview of research methods and ethics, the design of the M&E data collection activities, how to explain the purpose of the research to providers and what their role in the study would be, coaching providers on the register information to be filled out, and obtaining informed consent. The recruitment procedures were piloted in Lagos as part of the training.

Providers were visited from March to June 2016 by the team conducting the recruitment. In multi-provider facilities, interviewers were instructed to ask for the person in charge of providing contraceptive services. A total of 205 providers consented to participate as a recruitment site. Each consenting provider was administered a short questionnaire to capture basic information on the facility’s and/or provider’s background, such as urbanicity, name and multiple contact phone numbers, medical profession, and types of injectable contraceptives offered. This questionnaire was programmed in ODK (Open Data Kit) and the recruitment team was trained on following the question flow and entering data on a mobile device.

A dedicated patient register was printed out for each participating provider to record information for women to whom they sold or administered injectable contraceptives. We asked providers to record information for all injectables users in order to avoid overemphasis of DMPA-SC. The field team explained the instructions for keeping registers to enrolled providers, including writing down each instance of a sale of an injectable contraceptive (of any brand), noting the type of injectable (i.e., Depo Provera, Noristerat, Sayana Press), and if the client verbally agreed to be contacted later for a phone survey. For women who consented to be contacted for the phone survey, providers were asked to record the woman’s name and phone numbers (up to 3 contact numbers). Survey interviewers then regularly followed up with providers by phone (every 2-3 weeks) to retrieve the register information. Of the 205 enrolled providers, 127 gave client register data over the period from March to June 2016 and were compensated with a total of 1000 Naira (~US$5.00) for their participation. Reasons for providers’ not supplying any client information included electing not to fill registers, losing registers, forgetting to do so, and closing down of the business. From the 127 providers with register information, a total of 1,423 injectable contraceptive clients were documented, of whom 1,179 were given DMPA-SC. Of the DMPA-SC users, 994 consented to be called for the phone survey.

**D. Phone survey**

The phone survey questionnaire was designed to capture quantitative measures of DMPA-SC users’ sociodemographic background, past contraceptive use, awareness of and care-seeking for DMPA-SC, provider choice, interaction with the provider, their experience with DMPA-SC, and interest in participating in follow-up in-depth interviews and another round of the phone survey. Because the length of the phone-based survey was of particular concern, draft survey instruments were iteratively tested from January through March 2016. A small convenience sample of women consenting to participate in the exercise, and referred to the research team by Bees, were contacted via phone to test question length, flow, comprehension, and clarity over the mobile phone network. Pre-tests were conducted in both English and Yoruba. The final survey instrument was translated into Yoruba by members of the research team with separate members doing the initial translation and back-checking.

The survey was programmed in ODK and survey interviewers were trained over a three-day period. Training focused on how to appropriately and sensitively contact women over the phone, paying particular attention to protecting confidentiality, obtaining verbal informed consent, and asking survey questions effectively. Interviewers were also trained on using the survey program on mobile devices to enter in survey data, including skip patterns and making corrections.

Between March and September 2016, all consenting customers (*n*=994) were called to complete an initial phone survey administered by a trained, bilingual (in Yoruba, the dominant local language) interviewer. Up to five attempts were made to contact each potential respondent. For women completing the survey, the interview lasted about 15-20 minutes, and participants were compensated with 200 Naira (~US$1.00) of mobile phone credits. Of the 944 women called, 541 women completed the initial phone survey about their recent experience obtaining a dose of DMPA-SC; 374 women were not able to be reached after up to 5 attempts, 33 refused to participate, 22 were not eligible, and 24 phone numbers were incorrect. About half (*n*=266; 49.6%) were contacted within one month of their injection, 32.6% (*n*=175) were contacted within two months, 16.0% (*n*=86) within three months, and 1.7% (*n*=9) within 4-5 months.^[[2]](#footnote-2)^

About three months later, timed for after respondents were due for a reinjection (range 2.2-5.6 months; median 4.7 months), all 541 respondents who verbally consented to be contacted again during the initial phone survey were called to complete a second phone survey about care-seeking for a subsequent dose of DMPA-SC. The follow-up call lasted about 5-10 minutes, and participants were given 100 Naira (~US$0.29) of mobile phone credits for compensation.^[[3]](#footnote-3)^ In total, 342 women completed the follow-up phone survey.

Due to high rate of non-responses and potential systematic biases that result from the cascading sampling approach through providers referred by DKT Nigeria, we used two methods to assess the representativeness of our phone survey sample. First, we compared the sample characteristics to those of modern contraceptive users from 2013 Nigeria Demographic and Health Survey (NDHS), the only population-representative sample available at the time.[6] To further improve the comparability of these two data sources, we restricted the NDHS sample to only urban areas of the same 7 states where the M&E data collection occurred. While ideally, we also wanted to restrict the NDHS sample to only users of injectable contraceptives, the resulting sample size was too small to yield valid population-level inferences. Thus, we isolated the NDHS sample to the subset that were users of longer-acting, reversible methods (i.e., injections, IUD, and implants) in order to compare to phone survey respondents who had similarly had used a longer-acting, reversible method before switching to DMPA-SC. Sample weights were applied when extracting the user characteristics from the NDHS.

Second, because the resulting phone survey sample had so few younger women under age 25 or who were unmarried, we compared the subset who obtained DMPA-SC from a DKT Bee to complete customer registers from a sample of DKT Bees. DKT Bees were given customer registers to record all sales of DMPA-SC to women as part of their regular standard operating procedures. Because Bee registers recorded limited information about clients themselves, we were only able to compare the age and marital profile of all customers on Bees’ registers to the sample that completed the phone survey. We found no substantive differences in age or marital status between the percentage of phone survey respondents obtaining DMPA-SC from a DKT Bee and the complete list of clients listed in DKT Bees’ complete registers. In fact, when contacting some women additionally referred to the research team for user in-depth-interviews, a number of women under age 25 who were listed as unmarried were actually found to be married (see Section E), suggesting that errors in data recording on DKT Bees’ registers may overstate the percentage of young or unmarried women served.

**E. In-depth interviews**

*DMPA-SC users interview guide and sampling*

In-depth interviews were conducted with DMPA-SC users in order to provide data on areas of interest for the program M&E that could not be captured adequately through quantitative phone survey measures. Topic focus areas including user experience with DMPA-SC and decision-making around method uptake, switching, and continuation after the first dose. Draft interview guides were developed and revised between November 2015 and April 2016 based on emerging information from other M&E activities. A pre-test with consenting users from the phone survey was then conducted by the field team in Oyo state in May 2016, after which the guide was finalized in English. After translation and back translation by professional translators, the research team comprised of interviewers, transcribers, editors, coordinators, and supervisors met to review the guide in Yoruba and Pidgin. The group discussed areas of discord, and consensus was reached on a final version of the guide. The final interview guide included questions on the following topics: family background and contraceptive goals, experience in obtaining DMPA-SC (reasons for choosing the particular provider, interaction with the provider, and choosing among methods), reasons for switching to DMPA-SC if applicable, experience with DMPA-SC, and reinjection and intentions to continue the product’s use.

All respondents to the first round of the phone survey were asked if they were interested in participating in a longer, in-person interview. Those who responded affirmatively and were residing in Lagos, Oyo, or Ogun states (N=292), where the highest concentrations of phone survey respondents were located, were included in the sampling frame for the user IDIs. The sample was restricted to residents from the 3 selected states for logistical ease. We stratified these participants into three groups: never-married users, younger married users (age 24 or younger), and older married users (age 25 or older) based on program interest in reaching young or unmarried users. We also sampled for variation across the three states, the type of provider from which DMPA-SC was obtained, and whether the user was a new user or had switched from any other modern method of contraception. We selected 40 older married users, 23 younger married users, and 17 never-married users (which were all the consenting never-married and younger married users from the phone survey database in the 3 selected states).

From this total of 80 users, we aimed to complete 40 interviews. A round of screening and re-consent phone calls were first made with the selected 80 users prior to visiting them in-person. A large number of users, including 16 of the 17 never-married users, were unavailable (not reachable by phone) or refused the interview. Additional older married users were called in order to fill out this sample category; no additional never-married or young married users were available from the phone survey database. An additional list of younger, never-married users initially agreeing to be interviewed was submitted from Bee registers; 7 users on the list were selected for interview, but several were discovered to be actually married at the time of interview. A total of 42 user IDIs were completed: four with never-married users, 15 with younger married users, and 23 with older married users.

*DMPA-SC providers field guide and sampling*

In-depth interviews were similarly conducted with providers of DMPA-SC in order to better understand their views on and experiences with DMPA-SC, as well as their experiences with the social marketing program. As with the user IDI guide, a draft was developed in late 2015 based on the M&E objectives. Two pre-tests were then conducted, once in Osun state in February 2016 and another in Oyo state in May 2016. After the first pre-test, we decided to create a separate section of the guide specific to Bees due to the different nature of their service provision and engagement with DKT Nigeria. The final guide covered the following topics: contraceptive services provided and the local environment for contraceptives provision, views of and client demand for injectable contraceptives in general and DMPA-SC in particular, and contraceptive provision for unmarried versus married clients. For CHWs, the guide included an additional detailed section on their experience with program.

We stratified the 127 providers who provided user register information by the four main types of provider represented: (1) pharmacies, (2) proprietary and patent medicine vendors (PPMVs), (3) clinics, maternity homes, and hospitals (referred to as “health facilities” for brevity), and (4) Bees. Although we intended to additionally sample based on volumes of DMPA-SC clients, this was not possible due to the small number of providers who gave register data and low monthly volumes overall. For pharmacies, PPMVs, and health facilities, we therefore selected the 15 providers with the highest volumes of injectables overall (some of which did not sell any DMPA-SC during the period of observation, but had sold other injectable contraceptives). For Bees, we stratified by volume and selected a mix of those with relatively high, medium and low volumes. Interviews were successfully conducted with 57 providers: 14 pharmacies, 15 PPMVs, 15 health facilities, and 13 Bees.

*In-depth interview data collection and processing*

Interviews with providers and users were conducted by a bilingual (English and Yoruba) field team. The interviewers participated in a 3-day training on the study protocol, interview guides and ethics, overview of contraceptive methods, and in-depth interviewing techniques. The May 2016 pretests of both the provider and user guides were also conducted during the training, both to refine the guides and provide the team with feedback on their interview performance. Interviews with providers were conducted in August and early September 2016, after register data collection had ended, and typically lasted 40-60 minutes. Interviews with users were conducted in September and October 2016 and most lasted 30-40 minutes. Each user was only interviewed once for to complete an interview. Providers and users were given 500 Naira (~US$1.43) of mobile phone credits for their participation.

An administrative system was set up with a process monitoring tool that tracked each interview from the moment it was conducted. A naming convention was followed that indicated the category of person interviewed (provider, user), who conducted the interview and date. All interviews were digitally recorded, and transcribed either by a member of the field team or the supervisory team. Audio recordings of each interview were stored in our password-protected cloud-based server accessible only to our pool of translators and transcribers. Once translated and transcribed, each transcript was checked for errors and edited by a second person before being approved. Interviews conducted in Yoruba were simultaneously transcribed and translated into English.

**References**

[1] Nigeria Federal Ministry of Health. Task-shifting and Task-sharing Policy for Essential Health Care Services in Nigeria. Abuja: Federal Ministry of Health; 2014.

[2] Akeju DO, Vidler M, Sotunsa JO, Osiberu MO, Orenuga EO, Oladapo OT, et al. Human resource constraints and the prospect of task-sharing among community health workers for the detection of early signs of pre-eclampsia in Ogun State, Nigeria. Reprod Health 2016;13:111. doi:10.1186/s12978-016-0216-y.

[3] PATH. Sayana Press training materials. Seattle, WA: PATH; 2016.

[4] PATH. How to Introduce and Scale Up Subcutaneous DMPA (Sayana Press): Practical Guidance from PATH Based on Lessons Learned During Pilot Introduction. Seattle, WA: PATH; 2018.

1. Wholesale purchasers and associations were excluded as these entities were not directly involved in patient care. [↑](#footnote-ref-1)
2. An additional five women did not respond to the question of how long ago they received their injection. [↑](#footnote-ref-2)
3. Due to macroeconomic conditions, changes in the currency exchange rate affected the value of the pre-determined compensation for research participants starting July 2016. [↑](#footnote-ref-3)
